# A model of impaired Langerhans cell maturation associated with HPV induced epithelial hyperplasia

**DOI:** 10.1016/j.isci.2021.103326

**Published:** 2021-10-21

**Authors:** Zewen K. Tuong, Samuel W. Lukowski, Quan H. Nguyen, Janin Chandra, Chenhao Zhou, Kevin Gillinder, Abate A. Bashaw, John R. Ferdinand, Benjamin J. Stewart, Siok Min Teoh, Sarah J. Hanson, Katharina Devitt, Menna R. Clatworthy, Joseph E. Powell, Ian H. Frazer

**Affiliations:** 1 The University of Queensland Diamantina Institute, The University of Queensland, Woolloongabba, QLD 4102, Australia; 2 Australia Institute for Molecular Bioscience, The University of Queensland, St Lucia, QLD 4072, Australia; 3 Molecular Immunity Unit, University of Cambridge Department of Medicine, MRC-Laboratory of Molecular Biology, Cambridge, UK; 4 Wellcome Trust Sanger Institute, Hinxton, UK; 5 Garvan-Weizmann Centre for Cellular Genomics, Garvan Institute of Medical Research, Darlinghurst, NSW 2010, Australia

**Keywords:** Biological sciences, Immune system, Bioinformatics, Transcriptomics

## Abstract

Langerhans cells (LC) are skin-resident antigen-presenting cells that regulate immune responses to epithelial microorganisms. Human papillomavirus (HPV) infection can promote malignant epithelial transformation. As LCs are considered important for controlling HPV infection, we compared the transcriptome of murine LCs from skin transformed by K14E7 oncoprotein and from healthy skin. We identified transcriptome heterogeneity at the single cell level amongst LCs in normal skin, associated with ontogeny, cell cycle, and maturation. We identified a balanced co-existence of immune-stimulatory and immune-inhibitory LC cell states in normal skin that was significantly disturbed in HPV16 E7-transformed skin. Hyperplastic skin was depleted of immune-stimulatory LCs and enriched for LCs with an immune-inhibitory gene signature, and LC-keratinocyte crosstalk was dysregulated. We identified reduced expression of interleukin (IL)-34, a critical molecule for LC homeostasis. Enrichment of an immune-inhibitory LC gene signature and reduced levels of epithelial IL-34 were also found in human HPV-associated cervical epithelial cancers.

## Highlights

• Single cell atlas of Langerhans cells in cutaneous skin
• Stimulatory and inhibitory Langerhans cell states are in balance
• Inhibitory Langerhans cell states dominate HPV-transformed hyperplastic skin
• Langerhans cell imbalance is associated with disrupted IL-34 signaling

## Graphical abstract

**Figure:**
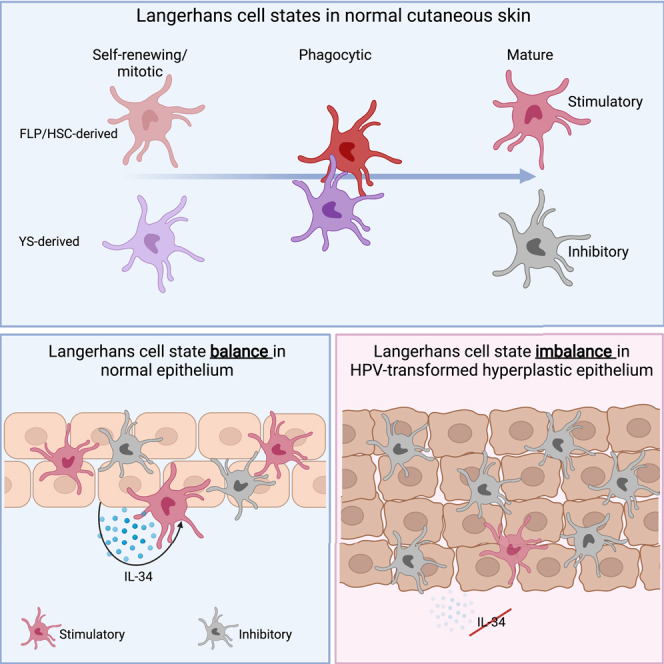

## Introduction

Langerhans cells (LCs) are an important subset of antigen-presenting cells (APCs) in cutaneous and mucosal epidermis, sharing both macrophage and dendritic cell (DC) phenotype and function, and regulating multiple aspects of barrier immunity (Doebel et al., 2017). The ontogeny of LCs has been studied in mice; LCs in cutaneous and mucosal tissues can arise from three sources: either yolk sac (YS)-derived precursors, embryonic fetal liver progenitors (FLP), or hematopoietic stem cell (HSC)-derived monocyte or DC precursors from the bone marrow (Hovav, 2018). The ontogeny of LCs varies between tissues; cutaneous LCs are comprised of YS-derived, FLP-derived and bone marrow HSC-derived monocytes, whereas mucosal LCs are comprised primarily of pre-DCs and monocytes from bone marrow HSC origins (Capucha et al., 2015; Iijima et al., 2007). In homeostasis, skin LCs undergo self-renewal (Merad et al., 2002) whereas mucosal LCs are replenished from circulating pre-cursors (Capucha et al., 2015). In human anogenital epidermis, LCs are distinct from CD11c^+^ CD1a epidermal DCs (Bertram et al., 2019). Despite these differences in origin, steady-state mucosal and cutaneous LCs express similar surface markers and are transcriptionally similar. All LCs express CD11b, CD11c, MHCII, Langerin (CD207) and EpCAM, and share common-gene expression patterns that are distinct from other tissue-resident APC populations, and are thought to be relatively homogeneous within their respective tissues (Capucha et al., 2015; Hovav, 2018; Ferrer et al., 2019). During acute inflammation, LCs migrate from the skin and the LC network is re-populated by monocyte-derived LCs (Ginhoux et al., 2006; Ferrer et al., 2019). The relative contribution of monocyte-derived LCs is still under debate, as both short-lived and long-lived monocyte-derived LCs have been reported after an acute inflammatory event (Greter et al., 2012; Sere et al., 2012; Wang et al., 2016; Chopin et al., 2013). Recent fate-mapping studies have shown that long-lived monocyte-derived LCs are transcriptionally virtually indistinguishable from embryonically seeded LCs after resolution of the inflammation (Ferrer et al., 2019).

Human papillomaviruses (HPVs) are epitheliotropic double-stranded DNA viruses that infect the basal keratinocytes of cutaneous and mucosal epithelium. Cervical and other anogenital cancers account for ∼5% of the global cancer burden (Torre et al., 2015) and are typically associated with infection by a subset of ‘high-risk’ HPVs. HPV16 and HPV18 are responsible for ∼70% of all cervical cancer cases worldwide and approximately ∼60% of oropharyngeal cancers are associated with HPV16 (Tang et al., 2013). Virus-like particle-based vaccines for cervical cancer have been highly effective in preventing infection with ‘high-risk’ HPVs (∼95%) (Frazer et al., 2011). However, although current HPV vaccines prevent HPV infection, they do not eliminate pre-existing HPV infections. HPV E6 and E7 viral proteins, which dysregulate DNA damage-induced cell death and cell cycle control, are candidates for therapeutic vaccines as they are expressed throughout the process of malignant transformation (Chandra et al., 2017). However, E6 and E7 proteins expressed in epithelia and epithelial cancers are poorly immunogenic. Further, the induction of immune responses to these proteins has, to date, failed as a therapeutic strategy in human HPV-associated cancers.

Because HPV infects squamous epithelium, where LCs are anatomically located and can cross-present exogenous antigens from the epithelium to stimulate antigen-specific T cell responses (Stoitzner et al., 2006; Holcmann et al., 2009), LCs are likely to be a key determinant of the host immune response to HPV. Although LC numbers are significantly lower and display reduced trafficking in HPV16-infected tissues (Caberg et al., 2009; Jiang and Xue, 2015; Kindt et al., 2016; Matthews et al., 2003), it is unclear why they fail to regulate immunity against HPV. A transgenic mouse model, K14E7, expressing the HPV16 E7 oncoprotein under the control of keratin 14 transcriptional promoter was developed as a model for chronic HPV16-infected epithelium. We have previously shown that K14E7 epidermis contains an altered myeloid cell composition, including the presence of CD103^+^ and CD11b^+^ DCs (Chandra et al., 2016). In addition, LCs in K14E7 epidermis express reduced levels of MHC class II, Langerin and Epcam, have reduced antigen-processing capacity and are less capable to facilitate T cell responses to intradermal delivered antigens (Bashaw et al., 2019, Bashaw et al., 2020; Chandra et al., 2016). Here we applied single-cell RNA-sequencing (scRNA-seq) to this model to better understand the extent to which dysregulation of the LC network is linked to the pathology of hyperproliferative skin expressing HPV oncogenes, and to identify whether LC interactions with keratinocytes are affected in the context of HPV-associated epithelial proliferation.

## Results

**Figure 1:**
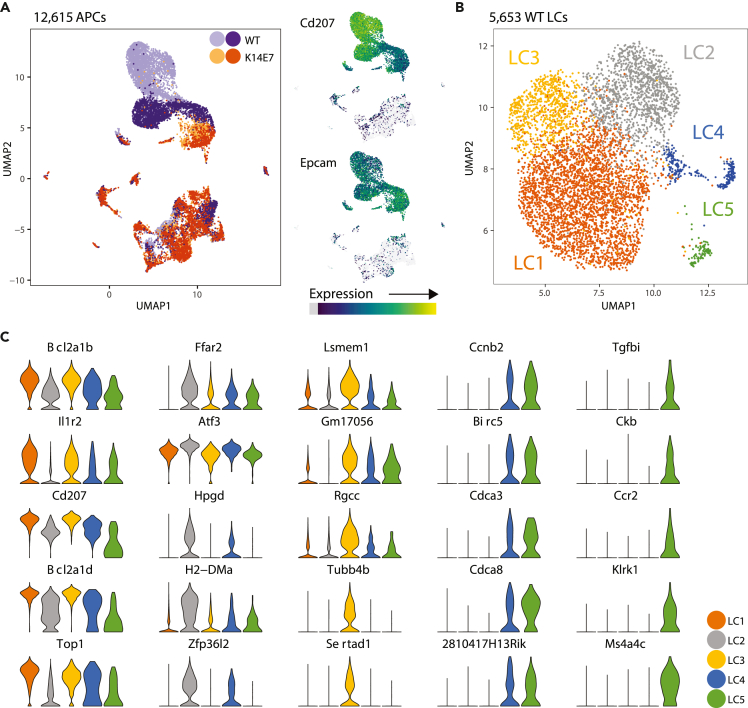
scRNA-seq of Langerhans cells (LC) sorted from epidermis and dermis of WT and K14E7 mice (A) (Left) UMAP plot of 12,615 cells identified as APC after initial QC and filtering. Purple dots indicate WT cells and orange dots indicate K14E7 cells, with the different shades denoting cells from replicate experiments. (Right) Expression *Cd207* and *Epcam* presented as a UMAP expression heatmap. Increasing expression is shown as a gradient from gray (zero expression), purple (low), blue/green (intermediate) to yellow (high). (B) UMAP plot of 5,653 WT LCs subjected to Seurat clustering. (C) Top five marker genes expressed by each LC cluster expressed as individual violin plots. Expression is scaled from 0 to 1 for each gene. See also Figures S1 and S2.

K14E7 transgenic mice develop epithelial hyperplasia characterized by an inflammatory cell infiltrate including diverse populations of myeloid cells (Figure S1A) (Chandra et al., 2016), and impaired local immune responses (reviewed in Bashaw et al., 2017; Bashaw et al., 2020; Bashaw et al., 2019). K14E7 epidermis contains, additional to CD11c^+^ Epcam^+^ LCs, also CD11c^+^ CD11b^+^ and CD11c^+^ CD103^+^ DCs, representing presumably cDC2 and cDC1 cells. To investigate the transcriptional characteristics of LCs in E7 transgenic skin, we performed 3′ scRNA-seq on live CD45^+^TCRβ^-^CD3ε^-^CD19^-^NK1.1^-^ cells from dermis and epidermis of non-transgenic C57BL/6 (WT) and E7 transgenic (K14E7) mice (Figure S1B). We defined a set of 12,615 cells as APCs based on expression of MHCII class II gene *H2-Ab1* (7,791 in WT and 4,591 in K14E7 skin) (Figures 1A and S1C). We observed groups of cells expressing the canonical marker genes for the major APC cell types, including LCs (Figure 1A), cross-presenting DCs, CD11b^+^ DCs, macrophages (Mac) and/or monocytes (mono) (Figure S1C).

### Langerhans cell transcriptomic heterogeneity in WT skin results from distinct immune, metabolic and cell cycle programs

We first generated a single cell atlas of skin-resident LCs in normal skin, using canonical correlation analysis (CCA) (Butler et al., 2018) on all WT APCs (batch 1: 4011 cells; batch 2: 3941 cells). The first 20 CCA components were aligned to account for batch effects (Figure S2A). Ten sub-clusters were identified from Louvain clustering implemented in Seurat (Figure S2B). CD11b^+^ DCs, CD8^+^ DCs, MHCII^+^F4/80^*int*^ or MHCII^+^F4/80^*lo*^ macrophages and Ly6c^−^/Ly6c^+^ monocytes (Figure S2C) were identified using a reference-based correlation analysis (Aran et al., 2019). LCs, marked by co-expression of Langerin/*Cd207* and *Epcam* (Figures 1A and S2D), constituted the majority (∼72%, 5,653 cells) of the total WT APCs and were considered in isolation and re-clustered, identifying five clusters (labeled as LC1 – LC5) (Figure 1B). The top five differentially expressed genes (DEGs) of LC1-5 were as follows: *Bcl2a1b*, *Il1r2*, *Cd207*, *Top1* and *Bcl2a1d* (LC1); *Ffar2*, *Atf3*, *Hpgd*, *H2-DMa*, and *Zfp36l2* (LC2); *Lsmem1*, *Gm17056*, *Rgcc*, *Tubb4b*, and *Sertad1* (LC3); *Ccnb2*, *Birc5*, *Cdca3*, *Cdca8* and *2810417H13Rik* (LC4); *Tgfbi*, *Ckb*, *Ccr2*, *Klrk1,* and *Ms4a4c* (LC5) (Figure 1C). These data suggest that LC1 to LC3 represent heterogeneous but related cell states, indicated by shared expression of DEGs at different levels across LC clusters. In contrast, LC5 expressed unique DEGs and clustered geographically distinct from LC1-3, suggesting that this LC cluster is significantly different. LC4 shared gene expression with both LC5 and LC1-3.

**Figure 2:**
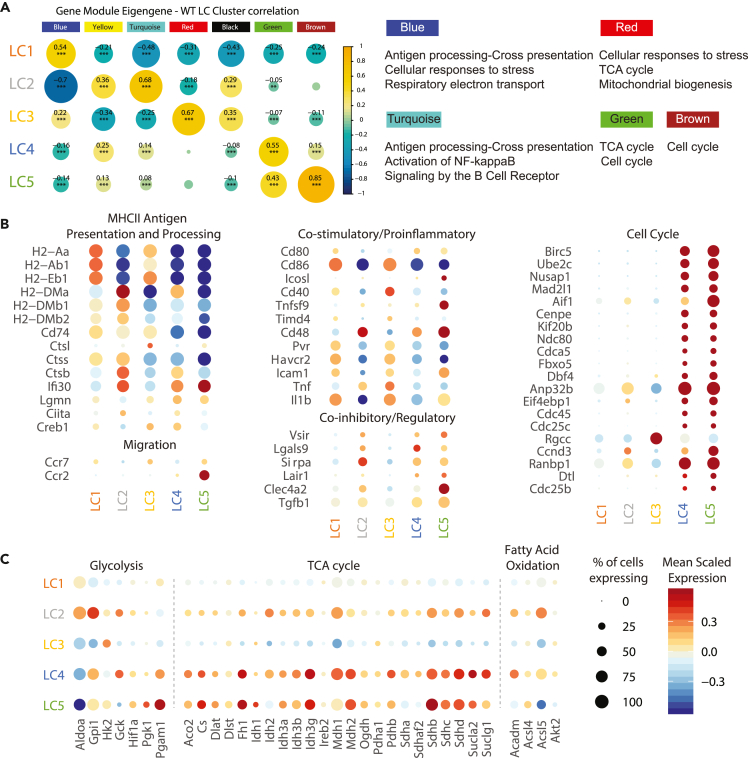
Distinct transcriptomic program of WT LCs (A) (Left) WGCNA analysis of WT LCs. Circle size corresponds to absolute module-cluster correlation score and circle color represents the correlation range from negative to positive correlation (blue to green to yellow). Modules displaying significant p values (after Benjamini and Hochberg correction) are denoted as follows: ∗p < 0.05, ∗∗p < 0.01, ∗∗∗p < 0.001. (Right) Summary of Reactome pathways associated with modules identified in WGCNA analysis. (B and C) Expression dot plots of genes grouped according to functions. Size of circles indicate percentage of cells expressing the gene (greater than zero), and color gradient corresponds to scaled expression level ranging from blue to white to red for low to mid to high expression across the LC clusters. See also Figures S3–S5.

To identify functional molecular networks in WT LC clusters, weighted gene co-expression network analysis (WGCNA) was performed, and network differential topology analysis used to identify gene modules (Langfelder and Horvath, 2008). Seven distinct gene modules (excluding a module containing unassigned weakly-correlated genes) showed distinct and significant cluster association (r > 0.5; B-H adjusted p value < 0.05) (Figure 2A). The modules that displayed the most significant positive correlations with the LC clusters were as follows: LC1 (blue); LC2 (turquoise); LC3 (red); LC4 (green); and LC5 (brown). Gene ontology enrichment analysis of the significantly co-regulated genes within each module was summarized as follows: blue (LC1) was associated with antigen processing and presentation, lymphocyte activation and immune signaling; turquoise (LC2) was associated with antigen-processing and cross-presentation, activation of NF-κB and regulation of cell metabolism; red (LC3) was associated with regulation of citric acid cycle and respiratory electron transport chain and cellular responses to stress; green (LC4) was associated with cell cycle and mitochondrial respiration; and brown (LC5) was associated with cell cycle (Figures 2A and S3A–S3E).

Examination of expression levels of mRNA encoding typical LC/DC markers and molecules demonstrated that LC1 and LC3 displayed higher expression of MHCII genes (*H2-Aa*, *H2-Ab1* and *H2-Eb1*), costimulatory molecules (*Cd86*, *Cd40, Pvr*/CD155, *Havcr2*/TIM-3, *Icam1*), and the proinflammatory cytokine *Il1b*, when compared to LC2, LC4, and LC5 (Figure 2B), indicating a mature phenotype. LC3 displayed the highest expression of these molecules, as well as the proinflammatory cytokine *Tnf* and MHCII-antigen processing lysosomal protease Cathepsin L (*Ctsl*). LC3 also expressed higher levels of the lymph-node homing *Ccr7* molecule, suggesting that LC3 represents a fully mature LC phenotype with capability to migrate. LC5 expressed the highest levels of the monocyte chemotaxis receptor *Ccr2* (∼50% of LC5 expresses the gene), and together with its cell cycle activity and absence of MHCII genes suggests this cluster represents a newly monocyte-derived emerging LC state (Figure 2B).

LC2 displayed the highest expression of genes encoding non-classical MHCII molecules (*H2-DMa*, *H2-DMb1,* and *H2-DMb2*), and genes involved in MHCII processing, including Cathepsins (*Ctss* and *Ctsb*), the lysosomal thiol reductase *Ifi30*, and transcriptional regulator of MHCII gene transcription (*Ciita*) (Figure 2B). Overall, LC2 appeared to express less co-stimulatory molecules and classical MHCII genes, with the exception of *Cd48*, indicating a semi-mature phenotype (Lutz and Schuler, 2002). LC2 together with LC4 and LC5 expressed more genes encoding co-inhibitory molecules (*Vsir*/VISTA, *Lgals9*/Galectin-9, *Sirpa*, *Lair1*/CD305, and *Clec4a2*/DCIR), when compared to LC1 and LC3. LC2, along with LC4 and LC5, also showed reduced expression of *Cd207* and *Il1r2* (Figure 1C).

LC4 and LC5 displayed the lowest expression of MHCII genes and *Cd86,* and the highest level of cell cycle related genes (Figure 2B). Cell-cycle phasing analysis (Scialdone et al., 2015) revealed that >99% of cells in LC1 – 3 were not dividing, whereas ∼25% and ∼50% of cells in LC4 and LC5, respectively, were dividing/proliferating cells (Figure S3F).

The LC clusters were also metabolically distinct, with LC2, LC4, and LC5 expressing higher levels of genes associated with glycolysis, TCA cycle and fatty acid oxidation, when compared to LC1 and LC3, consistent with an ‘energetic’ phenotype (Omenetti et al., 2019) (Figure 2C). LC3 expressed the highest level of Hexokinase (*Hk2*), a rate-limiting enzyme that controls the start of glycolysis, catalyzing conversion of glucose to glucose 6-phosphate. LC1 also expressed higher levels of phosphoglycerate mutase-1 (*Pgam1*) compared to LC2 and LC3, which catalyzes the conversion of 3-phosphoglycerate to 2-phosphoglycerate, also a rate-limiting step in glycolysis in phagocytes (Shalom-Barak and Knaus, 2002).

### Langerhans cell state heterogeneity in WT skin is associated with ontogeny

**Figure 3:**
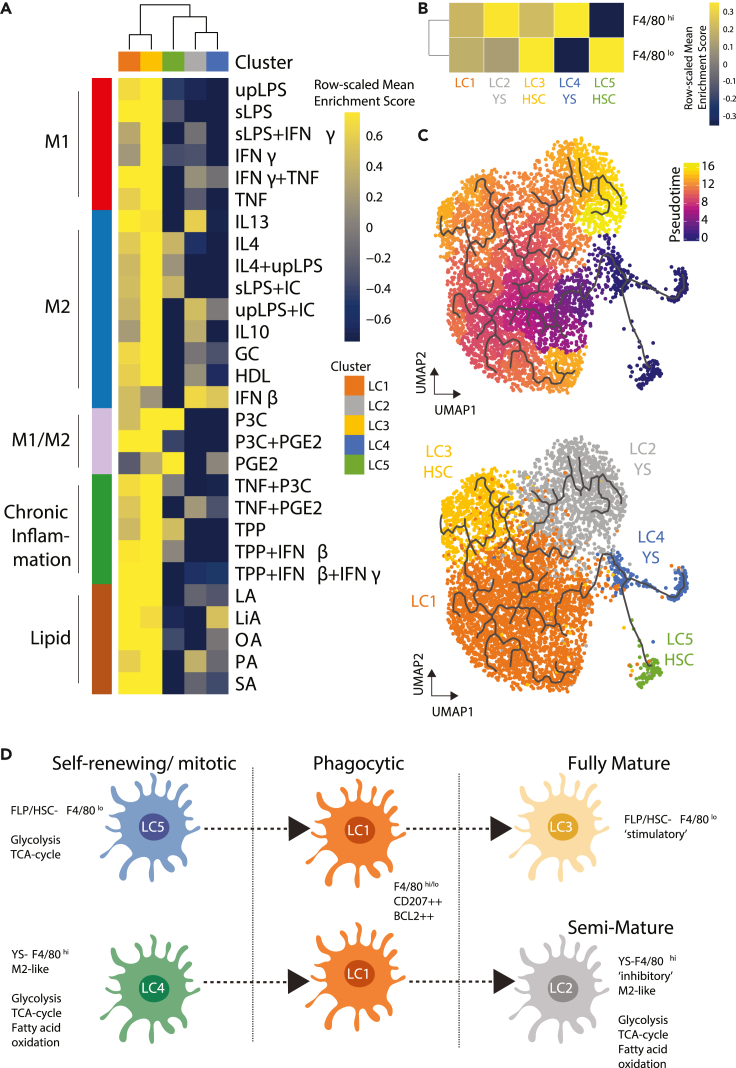
Polarization and trajectory analysis of WT LCs (A and B) (A) Gene set enrichment analysis for 28 macrophage stimulation transcriptomics signatures and (B) YS-F4/80^*hi*^ or FLP/HSC-F4/80^*lo*^ gene signatures. Enrichment is displayed as a heatmap corresponding to scaled mean enrichment score in each cluster. (C) Trajectory analysis of WT LCs using Monocle 3. Pseudotime ordering in the top panel is expressed as a heatmap from dark blue to purple to orange to yellow. Colors corresponding to the LC clusters are projected onto the trajectory in the lower panel. (D) Illustration of proposed model of LC cell state heterogeneity in WT skin. See also Figures S4 and S5.

The distinction in metabolic programs, as well as differential expression of costimulatory/inhibitory molecules, suggested that the different LC clusters may be variably polarized toward resting/M2-and/or activated/M1 states (Murray, 2017). Alignment of the LC cluster transcriptomes with that of 28 macrophage stimulation gene-sets corresponding to induction of variable macrophage polarization across a spectrum of activation states (Xue et al., 2014) revealed that LC3 displayed the relative highest enrichment of all stimulation signatures, suggesting a pan-activated state (Figure 3A). LC1 displayed a moderate enrichment of the same gene sets, suggesting that this cluster is less mature. In contrast, LC2, 4 and 5 displayed low enrichment for most stimulation gene sets with the exception of gene sets related to anti-inflammatory M2 and M2b (Huang et al., 2018). LC2, and LC4 to a lesser extent, were enriched for M2 (IL10, IL13, and IFN-β) and M2b (ultra-pure LPS + immune complex) gene sets, whereas LC5 was enriched for IL4, IL4+LPS and soluble LPS + immune complex gene sets (Figure 3A).

Fate-mapping of macrophages, including LCs, has previously established key transcriptional differences between YS-derived macrophages and those derived from FLP/HSC (Schulz et al., 2012). To determine whether the epidermal LC clusters identified in our data share transcriptomic similarities with YS or FLP/HSC ontogeny, gene set enrichment analysis was undertaken using gene sets curated from Schulz et al. (Schulz et al., 2012). We observed enrichment of YS-derived F4/80^*hi*^ associated genes in LC4 and of FLP/HSC-derived F4/80^*lo*^ associated genes in LC5 (Figure 3B), and this together with their expression of cell cycle genes (Figure 2B), suggests that LC4 and LC5 represent self-renewing LCs of different origins/lineage. LC1 were enriched for both lineage gene sets equally, whereas LC2 showed increased enrichment for YS-derived F4/80^*hi*^ and LC3 for FLP/HSC-derived F4/80^*lo*^ gene sets, suggesting their differentiation from LC4 and LC5, respectively (Figure 3B). To further explore the relationship between these LC populations, we performed cell fate trajectory inference, which suggested that mitotic LC4 and LC5 might give rise to phagocytic LC1 and develop into either LC2 or LC3 (Figure 3C). WGCNA analysis and information theory network reconstruction (Chan et al., 2017) of LC1 expressing genes revealed that the core hub genes included *Cd207*, *Il1r2*, and genes from the *Bcl2-*family including *Bcl2a1b*, *Bcl2a1d,* and *Bcl2a1a* (Figures S5A and S5B). The BCL-2 family encodes pro-survival/anti-apoptotic proteins (Schenk et al., 2017) and has been implicated in regulation of survival of different DC subsets (Carrington et al., 2015). In support of this, expression of the BCL-2 gene family in *Cd207*^*+*^ cells in WT epidermis was specifically confirmed (Figure S5C). Taken together, the results suggest that steady state skin contains both YS-derived (LC4) and FLP/HSC-derived (LC5) mitotic LCs, which give rise to transcriptionally similar phagocytic LCs (LC1), which develop further into two states of mature LCs with distinct transcriptional activity, either ‘anti-inflammatory/inhibitory’ YS-associated LC2 or ‘immunogenic/stimulatory’ FLP/HSC-associated LC3 (Figure 3D). We note that there is likely to be underlying heterogeneity/plasticity as both lineage-defining signatures are to a degree enriched in all three mature LCs clusters/states (Figure 3B).

### Langerhans cells in E7 expressing skin resemble dominantly an inhibitory mature cell state

**Figure 4:**
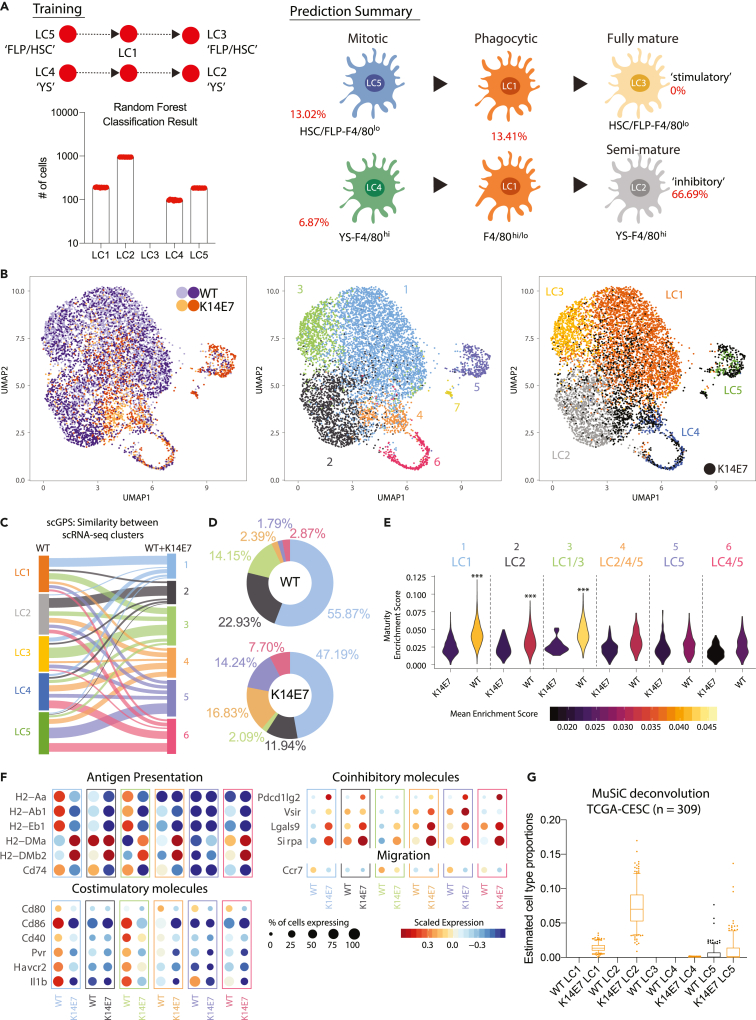
Analysis of LCs from K14E7 skin (A) Random forest classification of K14E7 cells with WT LCs used as training set. Each dot on the bar chart represents the result from 1 of 10 bootstrap runs. Classification results are summarized in the schematic (right), showing the percentage of K14E7 cells classified into the respective WT LC subtypes. (B and C) (B) (Left) UMAP plot of WT and K14E7 LCs after CCA alignment. (Center) 7 clusters were identified in the combined analysis. (Right) Original WT LC assignment superimposed on K14E7 cells (black) (C) scGPS analysis results presented as Sankey plot. Class assignment was trained using WT LC1-5 (left) and prediction was performed using WT + K14E7 aligned CCA clusters (right). Links between nodes correspond to transition/similarity scores predicted by scGPS. (D) Distribution of cells to the 7 clusters in WT and K14E7 samples presented as a pie chart. (E) Violin plot of AUCell gene set enrichment for genes up regulated by mature stimulatory DCs versus immature DCs (GSE9946) for each WT + K14E7 CCA aligned LC cluster. Mann-Whitney U test ∗∗∗p < 0.001. (F) Expression dot plot of genes grouped according to functions and split by WT or K14E7 cells. Size of circles indicate percentage of cells expressing the gene (greater than zero), and color gradient corresponds to scaled expression level ranging from blue to white to red for low to mid to high expression across the LCs. (G) MuSiC bulk-tissue cell-type deconvolution of n = 309 TCGA-CESC samples using the murine LC single-cell data. Boxes extend to 25^th^ and 75^th^ percentile while whiskers extend to 10^th^ and 90^th^ percentile. Line in the middle indicates the median. See also Figures S6 and S7.

Having characterized LCs through single cell transcriptomics in normal WT skin, we next explored how LC differed between normal skin and hyperplastic K14E7 transgenic skin. CCA alignment was similarly performed on two independent CD45^+^ K14E7 samples (batch 1: 1945 cells; batch 2: 2718 cells) and 11 APC clusters identified (Figure S6A), of which three could be classified as LCs (Figure S6B). In contrast to normal WT skin where LC comprised 70% of skin APCs, LCs constituted ∼30% of APCs in K14E7 skin (1,419 out of 4,591). This is in line with previous studies describing a diverse myeloid infiltrate in K14E7 skin additional to the presence of LCs (Bashaw et al., 2019; Chandra et al., 2016). To better understand the nature of the K14E7 LCs, a random forest classification was performed using the 5 WT LC clusters to train the classifier, resulting in >60% of K14E7 LC cells being classified as LC2, representing the semi-mature inhibitory YS-derived cell state, while no K14E7 LCs were assigned to the fully mature stimulatory WT LC3 cluster (Figure 4A). This data suggests that stimulatory fully mature FLP/HSC-derived LCs are absent in K14E7 skin, whereas inhibitory semi-mature YS-derived LCs are overrepresented.

To compare the WT and K14E7 LCs directly, we integrated the scRNA-seq data from K14E7 LCs (clusters 1, 7 and 11 (Figures S6A and S6B)) and non-transgenic LCs, which separated into seven clusters (Figure 4B). Cluster 7 was excluded from further analysis as they expressed epithelial gene markers and likely represented non-LCs. To test for a relationship between the six integrated WT + K14E7 LC clusters and the five WT LC clusters, the class probability of a cell belonging to a particular cell type was estimated using scGPS, a method which trains a penalized logistic regression model using marker gene expression (Nguyen et al., 2017). This approach showed that clusters 1 to 3 shared the largest similarities to WT LC1 – LC3 (mature LCs), while clusters 4–6 shared the largest similarities to WT LC4 and LC5 (self-renewing mitotic LCs) (Figure 4C). ∼92% of the WT LC were assigned to clusters 1 to 3, whereas only ∼59% of K14E7 LCs were assigned to the same clusters (Figure 4D). Similar to the random forest classification results in Figure 4A, K14E7 LCs were under-represented in cluster 3 (∼2.0% in K14E7 compared to ∼14.1% in WT), the cell cluster most similar to WT YS-F4/80^*hi*^ ‘stimulatory’ fully mature LC3 (Figures 4C and 4D). K14E7 LCs were instead overrepresented in clusters 4–6 compared to WT LCs (combined ∼37% in K14E7 compared to ∼7% in WT), the clusters most similar to the proposed ‘self-renewing/mitotic’ LCs (LC4 and LC5), indicating that the inflammatory microenvironment in K14E7 skin drives LC proliferation or recruitment of mitotic LC precursors (Figures 4C and 4D). Gene set enrichment analysis for maturity (Popov et al., 2008) demonstrated that WT LCs clusters 1–3 showed higher enrichment for maturity when compared to K14E7 LCs (Figure 4E). K14E7 LCs expressed lower levels of genes associated with classical MHCII antigen presentation (*H2-Aa*, *H2-Ab1*, *H2-Eb1* and *Cd74*), co-stimulation (*Cd80*, *Cd86*, *Cd40*, *Pvr*/CD155, *Hacvr2*/TIM-3), the proinflammatory cytokine *Il1b*, and migration (*Ccr7*), whereas non-classical MHCII molecules (*H2-DMa* and *H2-DMb2*) and co-inhibitory molecules (*Pdcd1lg2*/PD-L2, *Vsir/*VISTA, *Lgals9*/Galectin-9 and *Sirpa*) were up-regulated across all K14E7 LC clusters (Figure 4F).

To explore the clinical relevance of our observations, we performed bulk tissue cell-type deconvolution (Wang et al., 2019) of human cervical cancer biopsies held in The Cancer Genome Atlas (TGCA) and tested the LC cell-state and genotype-specific gene signatures for enrichment. Although we observed no enrichment of WT LC1-4 gene signature enrichment, cervical tissues (cancer and normal) were dominantly enriched with the K14E7 LC2 gene-signature, suggesting that LCs in the cervix adopt an inhibitory state (Figures 4G and S6C). Moreover, we observed a relative increase in LC4/LC5 enrichment in cancer and not healthy cervix (Figures 4G and S6C).

Taken together, our data show that the mature LC3 and fully mature stimulatory LC1 are under-represented and absent, respectively, in the K14E7 mouse model of HPV16 × 10^7^ induced hyperplastic epithelium. In contrast, LCs in K14E7 skin are either present as an early developmental state characterized by proliferation, or in an inhibitory semi-mature cell state. The inhibitory LC cell state was further enriched in human cervical data, even though here a gene signature of developmental LCs was enriched in cancer but not in healthy data, suggesting our findings are clinically relevant.

### Dysregulated Langerhans cell state is determined by epithelial hyperplasia

**Figure 5:**
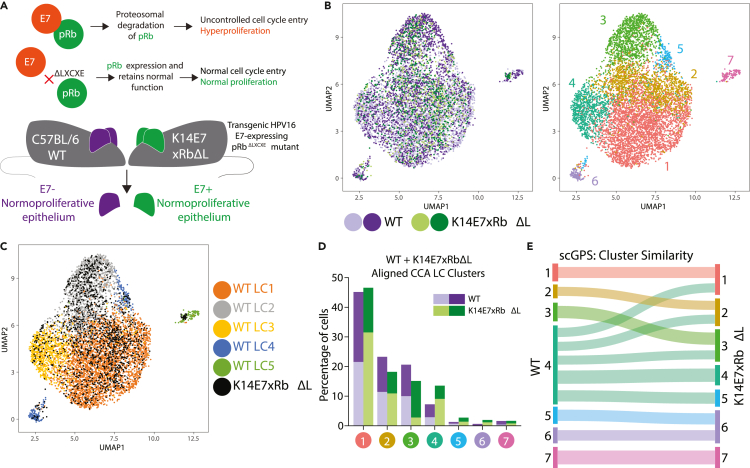
Hyperproliferation and not E7 induces altered LC cell states (A) Illustration of E7–pRb interaction and consequences, and K14E7xRbΔL mice. (B) UMAP plot of CCA aligned WT and K14E7xRbΔL LCs. Seven clusters are detected in the aligned CCA space. (C) Overlay of original WT LC1-5 assignment and K14E7xRbΔL cells (black). (D) Distribution of cells to the 7 clusters in WT and K14E7xRbΔL samples. (E) scGPS analysis results presented as Sankey plot. Class assignment was trained using WT cells (left) and prediction was performed using K14E7xRbΔL cells (right). Links between nodes correspond to transition/similarity scores predicted by scGPS.

To examine the molecular mechanism underpinning the transcriptional perturbation observed in K14E7 LCs, we hypothesized that E7's known interaction with retinoblastoma protein (pRb) (McLaughlin-Drubin and Munger, 2009) might directly lead to the altered LC composition (Figure 5A). We obtained scRNA-seq data from 2,125 CD45^+^ cells from epidermis of K14E7xRbΔL mice carrying a heterozygous or homozygous mutation at the E7-pRb LXCXE binding site (Balsitis et al., 2005). The major phenotypic difference in K14E7xRbΔL mice when compared with K14E7 mice is the absence of epithelial hyperplasia and loss of an immune cell infiltrate, despite the expression of E7 (Zhussupbekova et al., 2016; Choyce et al., 2013; Kuo et al., 2018). We integrated the WT and K14E7xRbΔL LCs, which separated into 7 clusters (Figures 5A and 5B). There was a substantial overlap between WT LCs and K14E7xRbΔL LCs (Figure 5C), indicating a high level of transcriptional similarity between the LCs in the two animal models. The proportions of LCs in each cluster were also similar between WT and K14E7xRbΔL mice (Figure 5D). scGPS analysis confirmed that WT LCs and K14E7xRbΔL LCs from the same cluster displayed high levels of transcriptional similarity, although cluster 4, which corresponds to WT LC3 (‘stimulatory’), displayed higher variability in the scGPS similarity scores (Figure 5E). Overall, the results from scRNA-seq of K14E7xRbΔL LCs support the hypothesis that epithelial hyperproliferation, conferred by E7-Rb interaction, is a major driver of LC alteration and aberrant polarization in the context of chronic HPV infection.

### Aberrant cell-cell communication determines Langerhans cells maturation in E7-induced hyperproliferative environment

**Figure 6:**
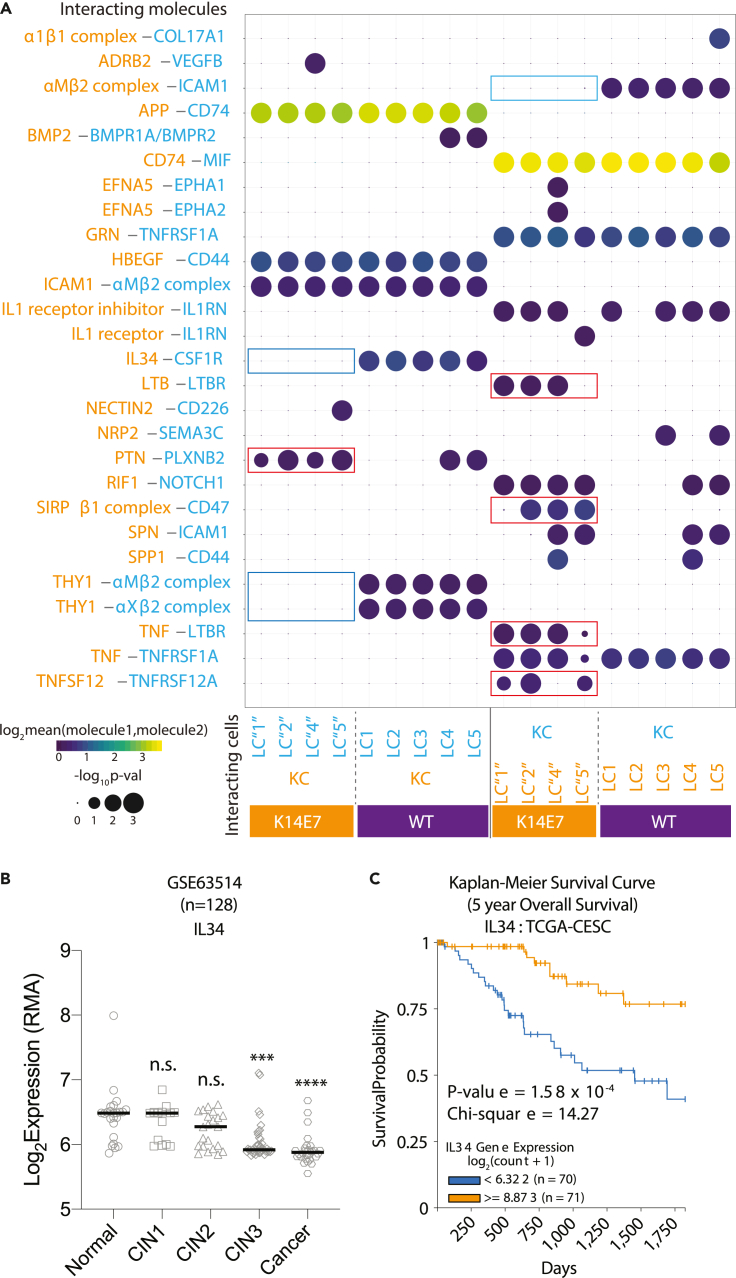
Aberrant cell-cell communication in K14E7 skin (A) CellPhoneDB analysis was performed for LC-KC pairs from WT or K14E7 animals. KCs in each animal group were pooled separately for the analysis. K14E7 cells were assigned to (WT) LC1, LC2, LC4 and LC5 based on random forest classification. For each statistically significant pair of molecules (p < 0.05), the mean expression values and associated p values were extracted. Circle size corresponds to -log_10_(p value) and the color gradient corresponds to Log_2_(mean expression values of each pair of molecules). Relevant molecule pairs are indicated by blue boxes (loss in K14E7) and red boxes (gain in K14E7). (B) Log_2_ gene expression values of *IL34* in 128 cervical cancer progression microarray dataset (GSE63514). Statistical tests were performed between normal versus CINs and cancer using Kruskal-Wallis test with Dunn's multiple comparisons correction in Prism where ∗∗∗p < 0.001 and ∗∗∗∗p < 0.0001. (C) Kaplan-Meier survival curve of 141 TCGA-CESC samples categorized as the top (orange) and bottom (blue) 25% percentile cohorts expressing *IL34*. Log rank test was performed where P = 1.58x10^-4^. See also Figure S7.

Hyperproliferation-related effects on LCs might result from aberrant cell-cell communication. Thus, we used CellPhoneDB, a tool developed to predict cellular interactions from scRNA-seq datasets, based on the expression of known receptor-ligand pairs (Vento-Tormo et al., 2018), to quantify receptor-ligand pairs differentially expressed by WT or K14E7 LCs and epidermal keratinocytes (KCs), using a scRNA-seq dataset previously generated from epidermal CD45-cells from WT and K14E7 animals (Lukowski et al., 2018) (Figure S7A). Interactions of LCs with KCs were of specific interest, as these cells are subject to hyperproliferation and are the most abundant cell type in the epidermis. This analysis identified a number of molecular interactions that are potentially dysregulated in chronic HPV infection, including some that were present in normal skin but absent in K14E7 skin and *vice versa*. Predicted interactions present in K14E7 LC and KC that were not identified in WT LC and KC included Pleiotrophin (PTN)-Plexin-B2 (PLXNB2), SIRPβ1-CD47, lymphotoxin beta (LTB)-LTB receptor, TNF-LTB receptor, and TNFSF12/TNF-related weak inducer of apoptosis (TWEAK). TNFSF12A/TWEAK receptor was present on K14E7 LCs and KCs respectively (Figure 6A), and was previously associated with poor survival outcomes when expressed at high levels (Budhwani et al., 2021).

Considering the interactions present between normal WT LC and KCs, but absent between K14E7 transgenic KC and LCs, all five WT LCs subtypes expressed integrin complexes of CD11b (aMb2) and CD11c (aXb2), that could interact with the CD90 (THY1) glycoprotein and ICAM1 expressed by KCs in normal skin (Figure 6A). However, this predicted interaction was absent in K14E7 LC, consistent with our previous observation that CD11b is downregulated on K14 × 10^7^ APCs (Figure S1A) (Chandra et al., 2016). Similarly, CSF1R-IL-34 interactions were identified between WT LCs with WT KCs, but not between K14E7 LCs and KCs (Figure 6A) (Lukowski et al., 2018). Indeed, there was significantly lower expression of *Il34* in K14E7 KCs (Figure S7B). IL34 is of particular interest, as it is critical for LC survival, with IL34-deficient mice lacking lymph nodes (LN) and demonstrating impaired skin immunity (Wang et al., 2012) (Lelios et al., 2020; Greter et al., 2012). Consistent with the mouse findings, *IL34* expression was significantly reduced in high-grade cervical intraepithelial neoplasia (CIN3) and cancer, but not normal or low-grade CINs (Figure 6B) (den Boon et al., 2015). The downregulation of *IL34* appears to be a general phenomenon associated with epithelial hyperplasia, as we could also observe a significant down-regulation of IL34 is psoriatic or eczema skin (Reynolds et al., 2021) (Figure S7C). We therefore hypothesized that a reduction in IL34 might be associated with poorer outcome in cervical cancer patients. In keeping with this, we found that cervical cancer patients expressing low levels of *IL34* have a significantly worse survival outcomes than those expressing high levels of *IL34* (Figure 6C), suggesting that disruption of IL34-mediated KC-LC interactions potentially delays LCs development (Figure 4G) and has a deleterious effect on anti-tumor immunity.

## Discussion

LCs are the primary antigen-presenting cell present in steady state skin, and their main role is microbe surveillance, the maintenance of peripheral tolerance toward friendly microbes and the initiation of immunity toward harmful intruders (Rajesh et al., 2019). The response LCs induce can be remarkably diverse and depends on the immunological context; LCs can facilitate antibody response, T effector responses, regulatory T cell responses, and even induce T cell anergy. It is reasonable to hypothesize that this functional diversity can be recognized at the transcriptomic level and in different immunological contexts, such as skin inflammation or skin cancer. Advanced technologies enable us now to delineate the transcriptional diversity at a single cell basis, and here we present the first single cell atlas of murine LCs in steady state and a model of hyperproliferative epithelium. We show that murine epithelial LCs can be categorized through transcriptomic analysis into groups with differing molecular genotypes likely to correlate with different functional phenotypes, and further show that hyperproliferative epithelium is associated with alterations in LC genotype that may explain poor immune responses to viral proteins expressed in hyperproliferative epithelium characteristic to HPV transformed epithelium.

LCs are located exclusively in cutaneous and mucosal epidermis in mice and humans, including the skin and cervical epithelium, and act as immune sentinels in these tissues, sampling the environment for antigens derived from normal tissues, infection or malignancy, to maintain immune homeostasis and to help initiate new immune responses (Hovav, 2018). Although there are some differences in the ontogeny of skin LCs and mucosal LCs, they are largely similar in phenotype and function (Capucha et al., 2015). Analysis of LCs in normal mouse skin using scRNA-seq identified two self-renewing/mitotic, one immature and two mature LC cell states, each characterized by distinct transcriptional programs. The transcriptional profile of the three non-proliferative LC states was most consistent with a steady-state LC expressing programs of antigen capture and two secondary states, one of which expressing antigen presentation programs, and the other representing a semi-mature state. By analogy with macrophage profiling, these LCs were pro-inflammatory or M1-like with a predicted capacity to stimulate adaptive immune responses, or anti-inflammatory, M2-like likely capable of inhibiting adaptive immune responses. The transcriptional profile also suggests that the adult skin LC network includes cells that arise from embryonically seeded YS-derived cells, as well as cells arising from FLP/HSCs precursors. Further, our analysis of the transcriptomic patterns associated with LCs of potentially differing origins suggests that the inflammatory/stimulatory and anti-inflammatory/inhibitory LC are derived from YS or FLP/HSC precursors, respectively, and could be traced back to mitotic YS and FLP/HSC precursors via trajectory analysis. Our data support the recent findings that cell types of both origins contribute to the network of long-lived LCs homeostatic skin (Ferrer et al., 2019). In contrast to this study (Ferrer et al., 2019), which showed that the replenished long-term LC from monocyte origins were indistinguishable from replenished embryonically derived LCs, our single-cell analysis approach enabled detection of YS-transcriptional imprinting versus HSC- transcriptional imprinting in semi-mature and fully mature LC subtypes and correlated LC origin with the different secondary cell states, whereas a different mature LC phenotype was transcriptionally indistinguishable for origin. Although it is still unclear whether there are functional differences in the steady-state and secondary LC cell states, the molecular features we identified at single-cell resolution enable further detailed profiling of these cell types.

LCs at barrier surfaces have access to skin pathogens, commensal organisms, allergens, contact sensitizers, and epidermal self-antigens (Hovav, 2018). Thus, LCs are presumed to mediate the initiation of adaptive immunity to foreign antigens and tolerance to self-antigens present in the skin. It has been proposed that the initial steps of immune evasion by HPV requires modulation of LCs (Da Silva et al., 2016; Fausch et al., 2002). LCs in HPV-infected hyperproliferative epithelium display characteristics of impaired antigen-presentation including reduced dendrites and reduced MHCII expression, indicative of an immature phenotype (Abd Warif et al., 2015; Chandra et al., 2016; Bashaw et al., 2019). More recently, we showed that LCs in the K14E7 skin displayed a significantly impaired ability to take up exogenous antigens, with no signs of activation after intradermal immunization with chicken ovalbumin (OVA) combined with a potent adjuvant QuilA. This impairment was correlated with a reduction in OVA-positive APCs in skin-draining LNs, diminished antigen-specific Th1 responses but increased regulatory T cells (Bashaw et al., 2019). The findings we report in this study support these earlier observations. In the current study, the ‘immunogenic/stimulatory’ fully mature FLP/HSC-derived LC cell state was virtually absent in K14E7 skin, despite an extensive immune cell infiltrate (Choyce et al., 2013). Rather, there was an increase in the YS-derived ‘anti-inflammatory/inhibitory’ semi-mature LC cell state. The E7 transgenic skin lacked the monocyte-derived LC development seen in response to acute inflammation (Ginhoux et al., 2006; Zhussupbekova et al., 2016), perhaps a consequence of failed replenishment or differentiation of the normal LC network. Cellular deconvolution also suggested expansion of LCs in developmental stages, which could become mature ‘inhibitory’ LCs in human cervical cancer. Previous therapeutic strategies in cervical cancer have attempted to non-specifically promote LC maturation, for example, by administering polyinosinic:polycytidylic acid (Poly I:C) (Da Silva et al., 2015, 2016). Because the development of macrophages/DCs/LCs depends on both intrinsic ontological cues and extrinsic environmental factors (Lin et al., 2015; Mass et al., 2016), including IL-34 signaling and RARa signaling (Hashimoto-Hill et al., 2018) for LCs, various LC states in our data are likely to be plastic and depends on provision of maturation signals from the environment. Our data suggest that perhaps manipulation of LC differentiation away from the trajectory toward the inhibitory LC state and instead toward a stimulatory LC trajectory may be more desirable. We note an important caveat in our study is that the tissue type of the skin (cutaneous) is fundamentally different from the cervix (mucosal), which can result in enrichment of different LC types (Bertram et al., 2019; Iijima et al., 2007; Merad et al., 2002), e.g., the However, we believe that the underlying immunological principles in hyperproliferative epithelium are still relevant for understanding immunological mechanisms during HPV-associated disease.

The majority of the immune dysfunction present in the K14E7 skin environment occurs in response to epithelial hyperplasia. Specifically, removing the ability of E7 to disrupt cell-cycle regulation via interaction with Rb in the K14E7xRbΔL mouse restored the LC phenotype to the WT. Our analysis also revealed that several important cell-cell interactions between LCs and KCs were down-regulated in K14E7 skin, including CSF1-R interaction with IL-34, which is required for optimal LC barrier immune functions (Wang et al., 2012), and Plexin-B2 interactions, implicated in positive regulation of macrophage motility (Roney et al., 2011). The lymphotoxin system has been implicated in positive regulation of DC homing, proliferation and homeostasis in spleen (Wang et al., 2005; Kabashima et al., 2005) and control of intracellular pathogen clearance by macrophages (Ehlers et al., 2003), whereas TWEAK has been implicated in tissue-reshaping processes during/after injury (Akahori et al., 2015) and high expression of TWEAKR contributes to worse survival outcomes in cervical squamous cell carcinoma and endocervical adenocarcinoma (CESC) (Budhwani et al., 2021). The interactions between LC and KC that are predicted not to occur in K14E7 transgenics may contribute to the previously observed loss of motility of APCs away from the E7-expressing skin to draining lymph nodes (Abd Warif et al., 2015). Importantly, the disruption of IL34-mediated keratinocyte-LC interactions may be clinically important in human cervical cancer, as low IL34 expression in cancer biopsies was associated with a worse prognosis. This axis may therefore be critical to promote LC anti-tumor functions and represents an interesting avenue for future research.

In conclusion, using a single-cell transcriptomic approach, we report here that normal mouse skin contains two LC cell ontogenies, each represented as diverse cell states on a developmental trajectory from proliferative to phagocytic to either fully mature/stimulatory or semi-mature/inhibitory. Aberrant LC transcriptomes were evident across all LC cell states in HPV16 E7-expressing skin, with an accumulation of semi-mature/inhibitory LCs and a complete absence of fully mature/stimulatory LCs. Epithelial hyperplasia was the major driver of this phenotype. We found that important cell-cell interactions required for LC differentiation and maintenance were disrupted in both HPV16 E7-expressing murine skin and human cervical cancer, including IL34-CSF1R, indicating clinical relevance. HPV16 E7 transgenic hyperplastic murine skin thus provides a robust model to investigate interventions to overcome impaired LC maturation induced by epithelial proliferation in HPV associated cancer.

Our work provides critical insights that will underpin future efforts to restore LC differentiation and maturation, cellular interactions and function in the treatment of chronic HPV-associated neoplasia.

### Limitations of the study

In this manuscript, apart from LCs, we have not performed comprehensive single-cell analysis of other antigen-presenting cells and cells from the lymphoid lineage. Although HPV16 E7 in K14E7 mice is expressed in keratin-14 + epithelial cells in the skin and cervical tissues (Ibarra Sierra et al., 2012), our single-cell analysis is restricted to cutaneous skin tissue, which is not the primary associated location of HPV16 burden in the context of cancer development (mucosa of the cervix and oropharynx). Further, development trajectories of LCs in cutaneous and mucosal sites are distinct (Capucha et al., 2015). Finally, the phenotypes displayed in K14E7 mice resemble pre-cancer rather than an invasive cancerous stage and these mice do not spontaneously progress; carcinogenesis in K14E7 mice requires additional carcinogens (Jabbar et al., 2010; Chung et al., 2008). For these reasons, additional studies are warranted to decipher LC cell state heterogeneity in mucosal pre-cancerous and cancerous disease models.

## STAR★Methods

### Key resources table

**Table:** 

REAGENT or RESOURCE	SOURCE	IDENTIFIER
**Antibodies**		

Anti-mouse CD16/CD32 (Fc block) (dilution 1:100)	BioLegend	Cat# 101301; RRID: AB_312800; Clone: 93
Anti-mouse CD45.2-PE-Cy7 (dilution 1:400)	BioLegend	Cat#109829; RRID: AB_1186103; Clone: 104
Anti-mouse TCRb-FITC (dilution 1:200)	BioLegend	Cat# 109205; RRID: AB_313428; Clone: H57-597
Anti-mouse CD19-FITC (dilution 1:100)	BioLegend	Cat# 115505; RRID AB_313640; Clone:6D5
Anti-mouse CD3e-FITC (dilution 1:100)	BD Biosciences	Cat# 553062; RRID: AB_394595; Clone: 145-2C11
Anti-mouse NK1.1-APC (dilution 1:200)	BioLegend	Cat#108709; RRID: AB_313396; Clone: PK136
Anti-mouse CD45-Percp-Cy5.5 (dilution 1:400)	BioLegend	Cat# 103131 ; RRID: AB_893344; Clone: 30-F11
Anti-mouse CD11c-PE-Cy7 (dilution 1:200)	BD Biosciences	Cat# 558079; RRID: AB_647251; Clone: HL3
Anti-mouse CD103-PE (dilution 1:100)	BioLegend	Cat# 121405; RRID: AB_535948; Clone: 2E7
Anti-mouse CD11b-AF700 (dilution 1:200)	BioLegend	Cat# 101222; RRID: AB_493705; Clone: M1/70
Anti-mouse CD326 (Ep-CAM)-APC (dilution 1:200)	BioLegend	Cat# 118213; RRID: AB_1134105; Clone: G8.8

**Chemicals, peptides, and recombinant proteins**		

Collagenase D	Merck/ Roche	11088858001
DNase	Merck/ Roche	11284932001
Dispase II	Merck/ Roche	04942078001
7-AAD Viability Staining Solution	ThermoFisher Scientific/ eBioscience	00-6993-50
DAPI	ThermoFisher Scientific/ Invitrogen	Cat# D1306; RRID: AB_2629482

**Critical commercial assays**		

Single Cell 3’ Library, Gel Bead and Multiplex Kit version 2	10X Genomics	PN-120233
NextSeq500/550 150-cycle High Output Reagent Kit version 2	Illumina	FC-404-2002
RNAscope® Multiplex Fluorescent Reagent Kit version 2	Advanced Cell Diagnostics	ADV323100

**Deposited data**		

K14E7 and C57BL/6 epithelial cells	(Lukowski et al., 2018)	ArrayExpress E-MTAB-6429
K14E7 and C57BL/6 CD45+ Lin- cells	This paper.	ArrayExpress E-MTAB-8199
Cervical cancer expression data	TCGAbiolinks (R/Bioconductor)	TCGA-CESC
Normal Cervix expression data	gtexportal.org	GTEx

**Experimental models: Organisms/strains**		

Mus musculus C57BL/6J-Tg(HLA-A2/H2-K)6Scr/HsdArc	Animal Resources Centre http://www.arc.wa.gov.au/	Product code: A2KB
Mus musculus K14E7	(Herber et al., 1996)	N/A
Mus musculus K14E7xRb^ΔL^	(Kuo et al., 2018)	N/A

**Oligonucleotides**		

RNAscope® 2.5 Probe - Mm-Cd207	In Vitro Technologies	ADV452521
RNAscope®Probe Mm-Bcl2a1b-C4 (Custom Probe)	In Vitro Technologies	ADV552531C4

**Software and algorithms**		

Kaluza	Beckman Coulter	https://www.beckman.com.au/flow-cytometry/software/kaluza; RRID:SCR_016182;
bcl2fastq	Illumina	v2.20
Cellranger	10X Genomics	v2.1.0
Seurat	CRAN	v2.3.4
WGCNA	CRAN	v1.70-3
Igraph	CRAN	v1.2.6
SingleR	Bioconductor	v1.6.1
ReactomePA	Bioconductor	v1.36.0
Enrichplot	Bioconductor	v1.12.2
AUCell	Bioconductor	v1.14.0
Fgsea	Bioconductor	v1.18.0
MuSiC	https://github.com/xuranw/MuSiC	v0.1.0
CellPhoneDB	https://github.com/Teichlab/cellphonedb	v2.0.5
Monocle 3	https://github.com/cole-trapnell-lab/monocle3	v1.0.0
scGPS	https://github.com/IMB-Computational-Genomics-Lab/scGPS	v1.6.0
NetworkInference	https://github.com/Tchanders/NetworkInference.jl	v0.1.0

### Resource availability

#### Lead contact

Further information and requests for resources and reagents should be directed to and will be fulfilled by the lead contact Ian Frazer, i.frazer@uq.edu.au.

#### Materials availability

This study did not generate new unique reagents.

### Experimental model and subject details

C57BL/6, K14E7, K14E7xRb^ΔL/+^ and K14E7xRb^ΔL/ΔL^ mice were maintained at the Translational Research Institute Biological Resources Facility (Brisbane, Australia). All mice were maintained under pathogen-free conditions, were female, and between 8-11 weeks of age when used in experimental procedures. All procedures were approved by The University of Queensland Animal Ethics Committee (UQDI/367/13/ NHMRC and UQDI/452/16).

### Methods details

#### Tissue collection and processing

Ear skin was split into dorsal and ventral parts and incubated floating epidermis side down in 2.5 mg/mL Dispase II (Merck/ Roche) for 1 hour at 37°C. Epidermis and dermis were separated with closed forceps. The dermis and epidermis were further homogenized by cutting into small pieces and digested separately with 1 mg/mL of collagenase D (Merck/ Roche) and 0.2 mg/mL of DNase (Merck/ Roche) for 1 hour at 37°C. Digested dermis and epidermis were passed through a 0.7-mm filter (BD Falcon, Franklin Lakes, NJ) and combined to generate a single-cell suspension for staining and sorting.

#### Cell sorting

Single-cell suspensions of digested epidermis + dermis samples were incubated with anti-mouse CD16/CD32 antibodies diluted in phosphate buffered saline for 30 minutes on ice to block Fc receptors. Samples were subsequently incubated with PE-Cy7-conjugated rat anti-mouse CD45.2 antibodies diluted in phosphate buffered saline plus 2% serum plus 2 mmol/L EDTA for 30 minutes on ice. Cells were further incubated with FITC-conjugated anti-mouse antibodies against lineage (Lin) markers CD3e, CD19, TCRb and APC-conjugated anti-mouse NK1.1 antibody. Before sorting, cells were labelled with 7-aminoactinomycin D (7-AAD). Live 7-AAD^-^CD45^+^Lin^-^ cells were sorted into 100% serum containing 2 mmol/L EDTA using the BD ARIA Fusion sorter (Franklin Lakes, NJ) at 12 p.s.i. with a 100-mm nozzle. Samples were kept chilled throughout the experiment prior to loading onto the 10X Chromium Instrument. Approximately 300,000 events/cells were collected per sample.

#### Flow cytometry

Epidermis and dermis single cell suspensions were analyzed using the following antibodies: PE-Cy7-anti-CD11c and PE-anti-CD103, PerCP-Cy5.5-anti-CD45, Alexa Fluor® 700-anti-CD11b and APC-anti-CD326 (Ep-CAM).

#### Single cell RNA-seq processing

scRNA-seq was performed in duplicate for cells sorted from pooled WT, K14E7 and K14E7xRbΔL transgenic animals. The 10X Genomics Chromium instrument was used to partition viable CD45+ cells with barcoded beads, and cDNA from each cell was prepared using the Single Cell 3’ Library, Gel Bead and Multiplex Kit (version 2, PN-120233; 10X Genomics) per the manufacturer’s instructions. Cell numbers in each reaction were optimized to capture approximately 5,000 cells. The resulting single-cell transcriptome libraries were pooled and sequenced on an Illumina NextSeq500, using a 150-cycle High Output reagent kit (NextSeq500/550 version 2, FC-404-2002; Illumina) in standalone mode as follows: 26 bp (read 1: 16 bp cell barcode plus 10 bp UMI), 98 bp (read 2: insert), and 8 bp (i7: sample index). scRNA-seq libraries were generated from two independent experiments, in which cells were pooled from 2-3 age-matched littermates in each experiment. From WT and K14E7, a total of 518,487,996 cDNA reads were obtained, with a mean of 29,509 reads per cell for 17,570 cells. A median 1,487 genes per cell were detected, which corresponded to 5,178 unique molecular identifiers (UMIs) per cell.

#### Single molecule RNA-FISH and Microscopy

Fresh-frozen, OCT-embedded tissue was cryosectioned at 8 μm and fixed in 4% PFA. RNA-FISH was performed using the RNAscope® Multiplex Fluorescent Reagent Kit v2 (Advanced Cell Diagnostics, Newark, CA) as per the manufacturer’s guidelines. In brief, fixed tissue sections were dehydrated with an ethanol series of increasing concentration and treated with hydrogen peroxide and protease before binding the sequence-specific RNA probes. A three-tiered amplification step enabled detection of low-abundant signal, while specific HRP-probes provided specific binding of OPAL fluorescent probes to the RNA probes. Slides were imaged with the PerkinElmer Vectra 3 Spectral imager at 20x magnification. Image exposure was normalized with InForm software.

#### Bioinformatic analysis

##### Bioinformatics preprocessing

Raw BCL files were processed using *cellranger* (v2.1.0; 10X Genomics) to create fastq files (*mkfastq*), individual count matrices (*count*) and aggregated datasets (*aggr*) with default parameters. The expected number of cells was set to 5,000 cell per sample. Reads were aligned to a custom reference genome comprising the mouse (mm10) and the HPV16 genome (NC_001526) using the STAR aligner (Dobin et al., 2013) included in the *cellranger* pipeline. High quality cell barcodes and unique molecular identifiers were retained and between-sample normalized gene expression matrices were generated using *cellranger aggr*.

The expression data generated by *cellranger* was used as the input for the Seurat analysis software (version 2.3.4) (Butler et al., 2018). Expression levels for each transcript were determined using the number of UMIs assigned to each transcript. Outlier cells and genes were filtered such that (i) only genes expressed in three or more cells (ii) cells expressing 200-2,500 genes, and (iii) cells expressing less than 10% mitochondrial genes were retained for analysis. Between-cell normalization was performed using the *LogNormalize* function with a scaling factor of 10,000, and cell-cell variation attributed to number of UMIs, and mitochondrial gene expression was regressed out. Seurat v2.3.4 canonical correlation analysis (CCA) procedure was used for batch correction (Butler et al., 2018); the first 20 CCA components were aligned to account for batch effects. scRNA-seq data was visualized using Uniform Manifold Approximation and Projection (UMAP) (McInnes et al., 2018) calculated from the aligned CCA-reduced expression data.

Cell types were initially classified by differential expression analysis comparing each cluster against all others with the Wilcoxon method in Seurat. Genes were considered significant if the adjusted P-value was below the multiple-testing threshold of 0.01 (Benjamini-Hochberg method) and the absolute log expression fold change was 0.5 or greater. A second stage of cell type classification was performed using SingleR (Aran et al., 2019). Cell cycle phases were predicted using the *cyclone* cell cycle scoring method implemented in *scran* (Lun et al., 2016; Scialdone et al., 2015).

##### Cell fate trajectory analysis

Monocle 3 (Cao et al., 2019) was used to reconstruct a pseudotime trajectory based on the UMAP embeddings of the LCs. The root of the trajectory was set on vertices nearest to LC4 and LC5.

##### Cluster similarity scoring

The LASSO regression procedure implemented in scGPS (https://github.com/IMB-Computational-Genomics-Lab/scGPS) (Nguyen et al., 2017) was used to calculate the deviance coefficient for each of the genes defining each LC population. For the scGPS input, we used the significant differentially expressed genes that were obtained by comparing cluster *x* to the remaining clusters, a vector of cluster information for each cell, and log_2_(counts + 1)-transformed expression data. Random forest classification was performed using the *ClassifyCell* function implemented in Seurat.

##### Weighted gene co-expression network analysis (WGCNA)

To establish LC gene co-expression networks, we applied weighted gene co-expression network analysis as implemented in the WGCNA package for R (Langfelder and Horvath, 2008), using 1352 genes found to be statistically significant between the WT subclusters with a requirement that the gene must be expressed by at least 25% of all cells. Only significantly highly correlated gene-gene networks were retained for further analysis.

##### Community analysis

Graph networks were reconstructed based on edges connecting genes, with weights from WGCNA analysis. Edges were filtered to remove those with weights lower than the 3^rd^ quartile of all edges. Nodes were also filtered based on a diagnostic plot of node degree distribution. To detect community structure of the networks, where related nodes are clustered into a dense subgraph, we applied a fast, greedy optimization of modularity (clustering) algorithm implemented in *igraph* R package (Clauset et al., 2004). Communities and nodes are colored in the network graph by membership identified from the clustering step.

##### Mutual information network analysis

To find key genes in a set of significant genes identified from WGCNA analysis of gene modules (blue module), we reconstructed a gene expression network based on a mutual information algorithm, which uses Empirical Bayesian model and Information theory (Partial information decomposition algorithm) (Chan et al., 2017). We also utilized the CellPhoneDB database of curated ligand-receptor interaction to set priors for the Bayesian network reconstruction (Vento-Tormo et al., 2018).

##### Pathway analysis

For each gene module of each subpopulation, a set of significant genes from the WGCNA were selected, such that P values for gene significance and module membership adjusted by Bonferroni correction were less than 0.05. We then performed Reactome pathway analysis was performed using the *ReactomePA* package (Yu and He, 2016). The Reactome database contains a manually curated, high-quality protein functional networks. The linkages of genes and Reactome categories were displayed in a gene-concept network plot implemented in the *enrichplot* package (Yu et al., 2010).

##### Gene set enrichment analysis

Single-cell gene set testing was performed using *AUCell* (Aibar et al., 2017) or *fgsea* Bioconductor R packages. Genes sets from the respective studies were downloaded from ArrayExpress (F4/80 signature - E-MEXP-3510 (Schulz et al., 2012)), MSigDB (Hallmark gene set collections (Liberzon et al., 2015) and mature DC gene set (GSE9946_IMMATURE_VS_MATURE_STIMULATORY_DC_DOWN) (Popov et al., 2008)), and GEO Omnibus (GSE47189-macrophage polarization signatures (Xue et al., 2014).

##### Cell type deconvolution

Bulk tissue cell type deconvolution of The Cancer Genome Atlas (TCGA)-CESC and Genotype-Tissue Expression (GTEx) RNA-seq data (n=11) was performed using MuSiC (Wang et al., 2019) with the normalized counts of the LCs in WT and K14E7 samples. K14E7 LC1-5 (excluding 3) were defined based on random forest classification results.

##### Kaplan-Meier survival analysis

Survival analysis was performed using the UCSC Xena Functional Genomics Explorer (https://xenabrowser.net) (Goldman et al., 2020). Using log2-transformed HTSeq count data (log2(count + 1)) from 141 TCGA primary cervical and endocervical squamous cancer (CESC) samples, a 5-year (1825 day) Kaplan-Meier overall survival curve was derived using quartiles of IL34 expression. Statistical significance of the 5-year survival probability of patients with increased expression of IL34 compared to those with lower expression was determined by log-rank test (chi-square).

### Quantification and statistical analysis

All quantification and statistical analysis methods and definitions are described in the associated figure legends and methods and were performed in the R statistical package.

## Data Availability

RNA sequencing datasets were deposited in ArrayExpress and are available via ArrayExpress: https://www.ebi.ac.uk/arrayexpress/experiments/E-MTAB-6429/ and https://www.ebi.ac.uk/arrayexpress/experiments/E-MTAB-8199/. This paper also analyzes existing, publicly available data. These accession numbers for the datasets are listed in the key resources table. This paper does not report original code. Any additional information required to reanalyze the data reported in this paper is available from the lead contact upon request.
