# Supplementary material for: A model of impaired Langerhans cell maturation associated with HPV induced epithelial hyperplasia
Source: iScience. 2021 Oct 21;24(11):103326. doi: 10.1016/j.isci.2021.103326 (PMC8586807; doi:10.1016/j.isci.2021.103326)
Supplement: Document S1. Figures S1–S7 [file mmc1.pdf]

iScience, Volume *24*

Supplemental information

A model of impaired Langerhans cell maturation associated with HPV induced epithelial hyperplasia

Zewen K. Tuong, Samuel W. Lukowski, Quan H. Nguyen, Janin Chandra, Chenhao Zhou, Kevin Gillinder, Abate A. Bashaw, John R. Ferdinand, Benjamin J. Stewart, Siok Min Teoh, Sarah J. Hanson, Katharina Devitt, Menna R. Clatworthy, Joseph E. Powell, and Ian H. Frazer

SUPPLEMENTAL INFORMATION

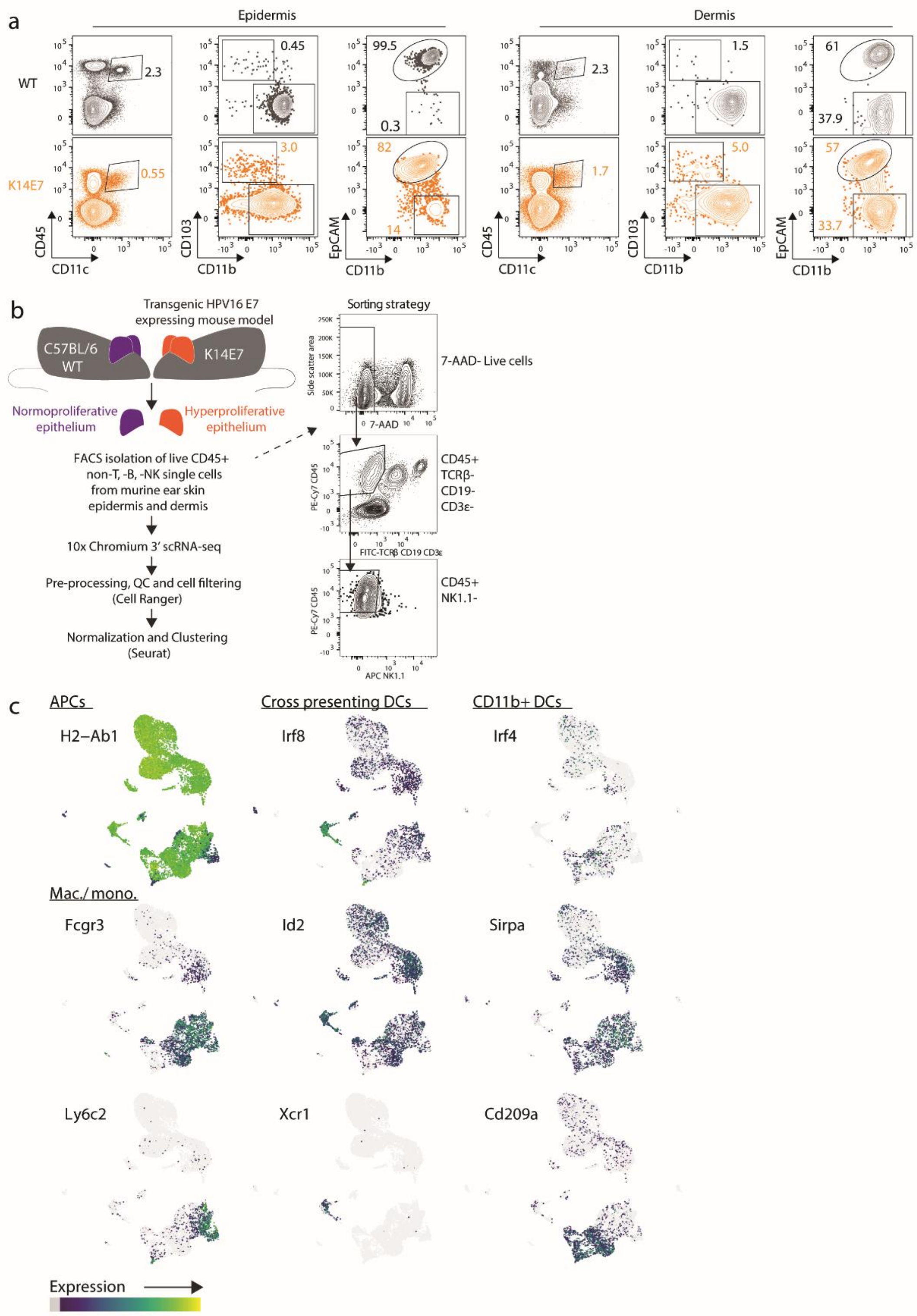

Figure S1. Related to Figure 1.

(a) Flow cytometry plots of DCs and LCs in dermal and epidermal single-cell suspensions of WT and K14E7 skin. LCs are marked by CD45+CD11b+CD11c+CD103-EpCam+ staining profile. (b) Flow cytometry sorting and bioinformatics work-flow of scRNA-seq experiment. Sorted 4-5x10^4 CD45+ live cells were pooled from 2-3 littermate pairs of WT and K14E7 mice (all female at 8-11 weeks old) and used as input for the 10X Genomics Chromium 3' gene expression kit (v2). The entire experiment was repeated independently. (c) Expression heatmap of known APC marker genes superimposed on UMAP plot. Increasing expression is shown as a gradient from grey (zero expression), purple (low), blue/green (intermediate) to yellow (high).

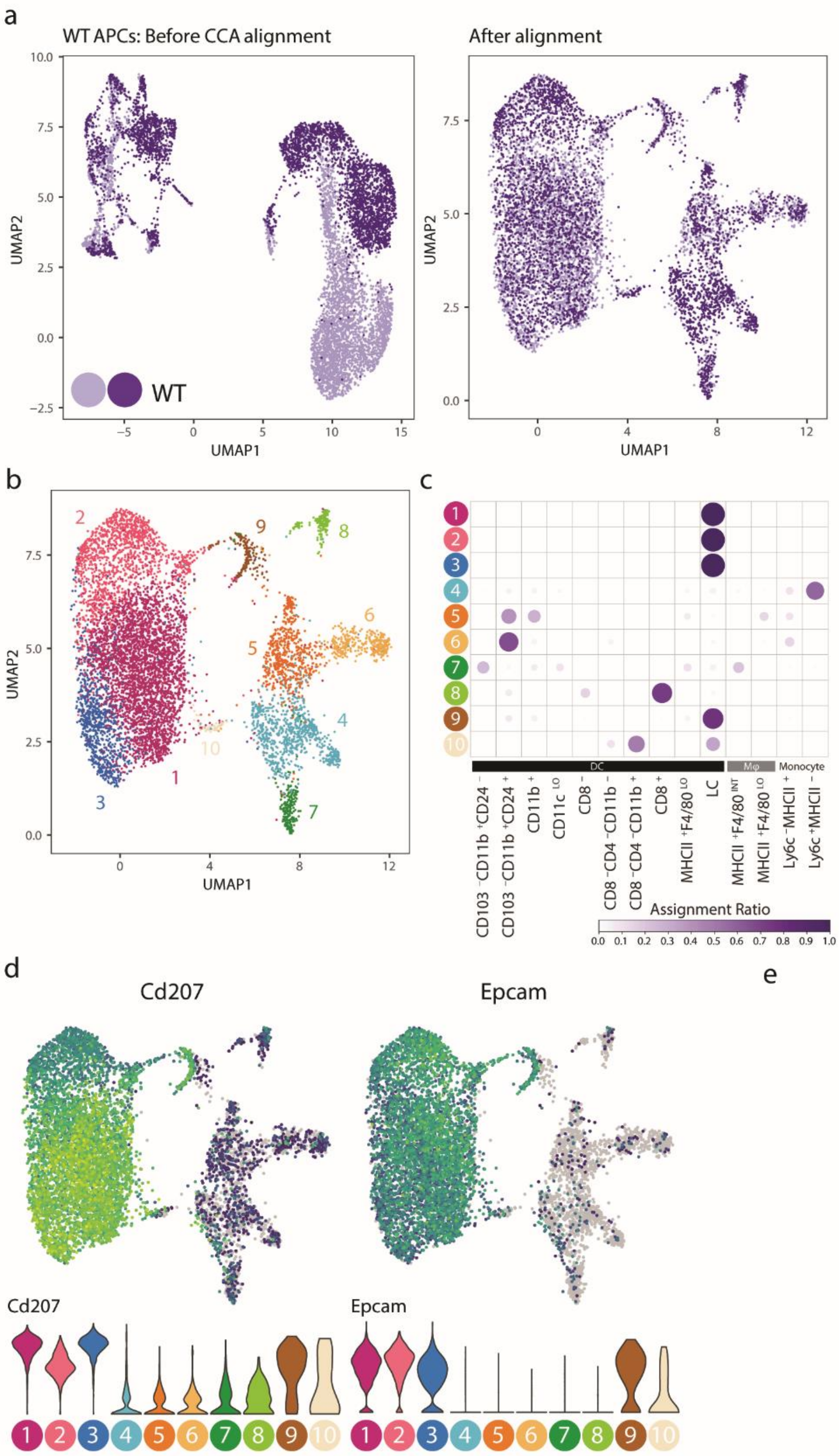

Figure S2. Related to Figure 1.

(a) UMAP plot of WT APCs before and after CCA alignment. The two shades of purple indicate the respective scRNAseq data from samples obtained from two independent experiments. (b) Clusters identified from Louvain clustering. (c) SingleR analysis of WT APCs. Size and gradient of circles indicate the proportion (ratio) of cells assigned to the various cell types within each cluster. The ImmGen database was used as the reference database for the analysis. (d) Expression Cd207 and Epcam presented as a UMAP expression heatmap (top) and violin plot (bottom). Expression is scaled from 0 to 1 for each gene.

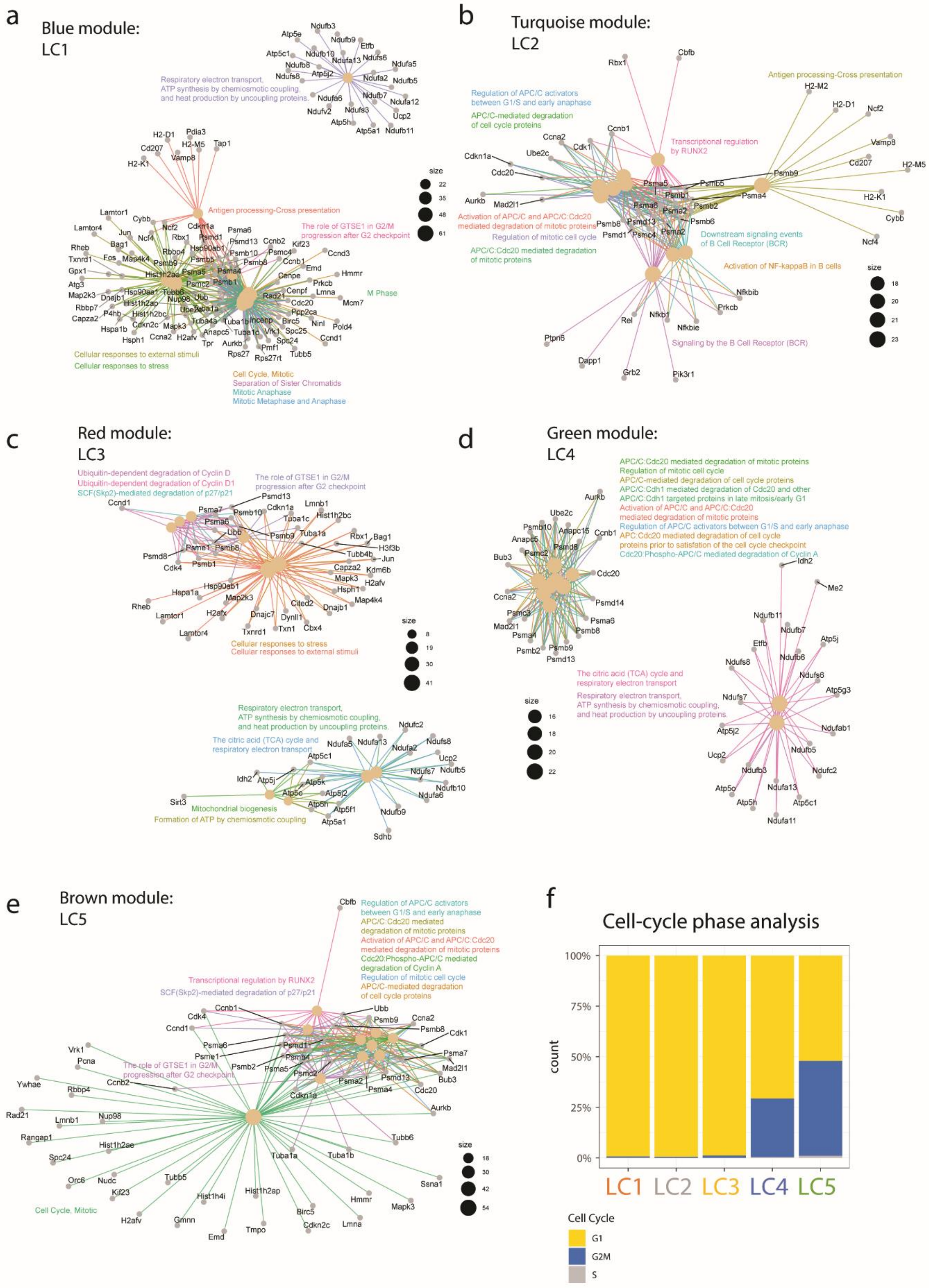

Figure S3. Related to Figure 2.

(a-e) Reactome pathway analysis of significant genes from WGCNA modules. Each network is specific for each gene module and the linkages of genes and Reactome categories were displayed in a gene-concept network plot implemented in *enrichplot* R package. (f) Cyclone cell-cycle phasing analysis results of WT LC1-5. Percentage of cells within each cluster assigned to a predicted cell cycle stage (G1, S and G2 phases) is displayed.

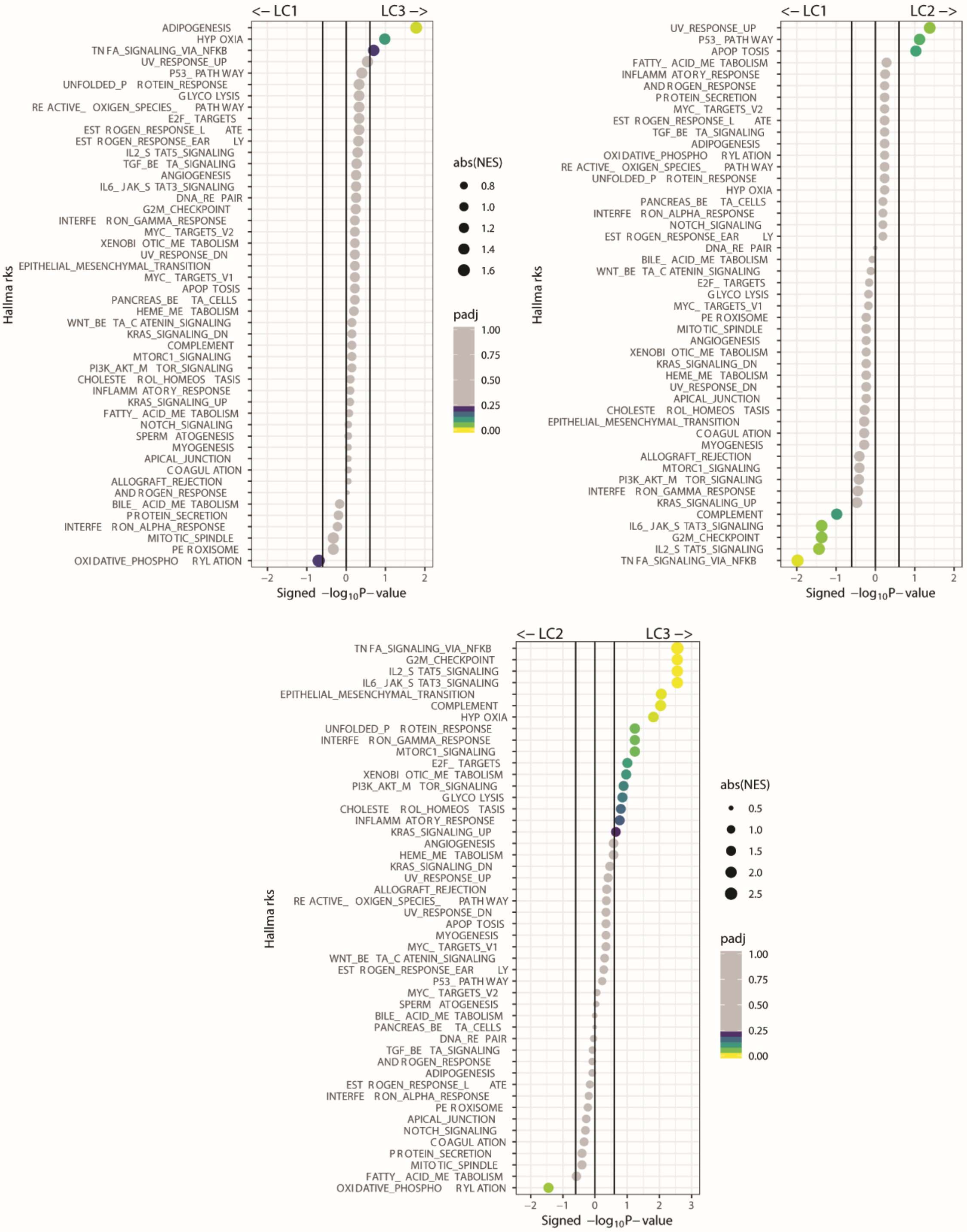

Figure S4. Related to Figure 3.

Gene set enrichment analysis of MSigDB Hallmark signatures using the differentially expressed genes between LC1 and LC3, LC1 and LC2, and LC2 and LC3. The pathways on the y-axis are ordered according to the magnitude of the signed -$\log_{10}$P-value (as shown on the x-axis). Additional vertical black lines flanking x = 0 (p-value of 1) mark $p < 0.05$. Size of circles correspond to absolute normalized enrichment score and the colour gradient indicates pathways that achieved adjusted p-value < 0.25.

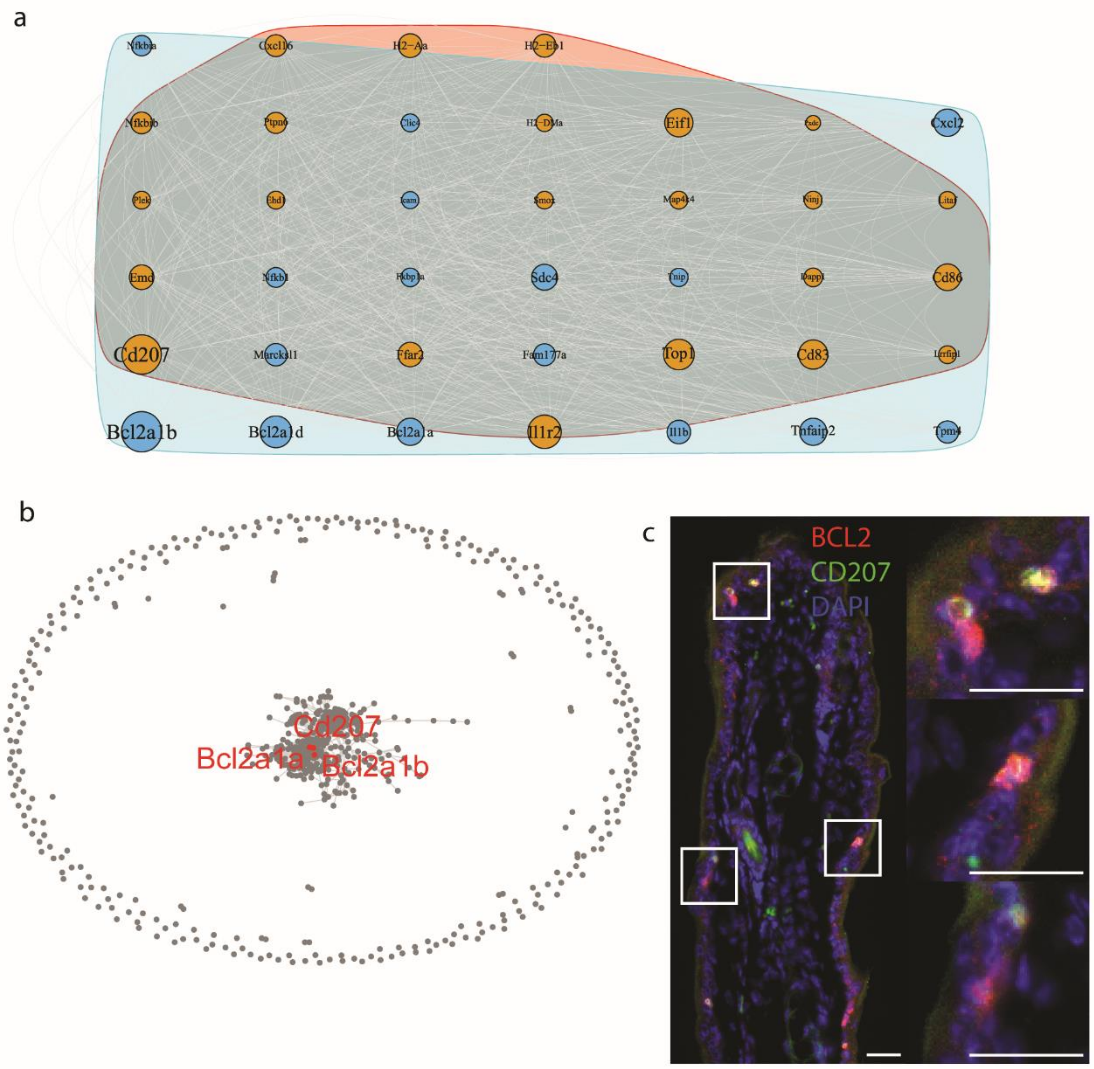

Figure S5. Related to Figure 2.

(a) Gene network community analysis for the blue module. Each identified community is a dense subnetwork with the higher density of edges within than between communities, suggesting potential coregulated genes. Size of nodes indicate node degree and edges are weights from WGCNA analysis (correlation strength). Colours for nodes and the background mark the gene community assignment. (b) Mutual information network analysis of the blue module. Nodes represent genes and edges indicate strength of mutual information in gene expression (using partial information decomposition algorithm). Key relevant hub genes with most connected edges are highlighted in red. (c) Single-molecule RNA-FISH image of WT skin stained for probes targeting murine BCL2 (red; *Bcl2a1b*) and CD207 (green; *Cd207*). Cell nuclei are stained by DAPI (blue). Scale bars = 50 μm.

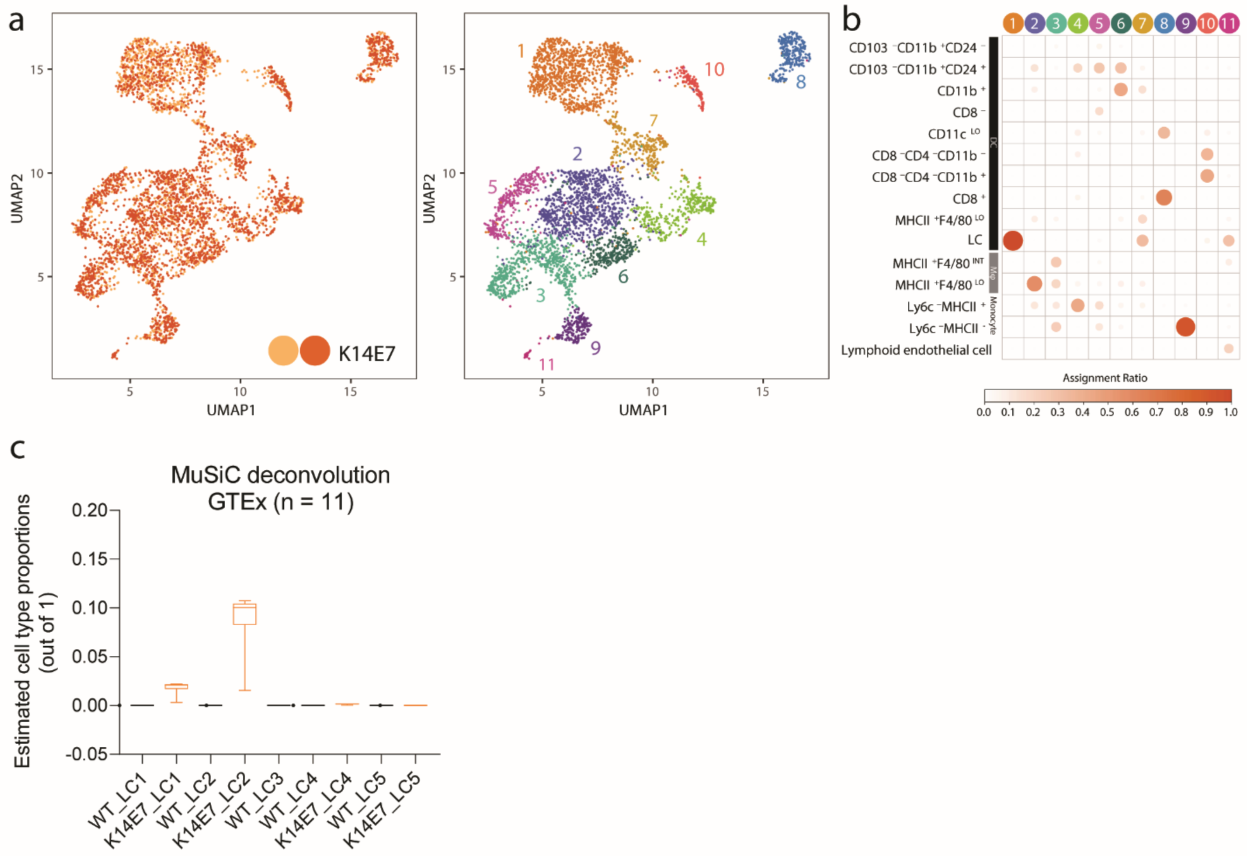

Figure S6. Related to Figure 4.

(a) UMAP plot of K14E7 APCs after CCA alignment and clusters identified by Louvain clustering. (b) SingleR analysis of K14E7 APCs. Size and gradient of circles indicate the proportion (ratio) of cells assigned to the various cell types within each cluster. (b) The ImmGen database was used as the reference database for the analysis. (c) MuSiC bulk-tissue cell-type deconvolution of n=11 GTEx samples using the murine LC single-cell data.

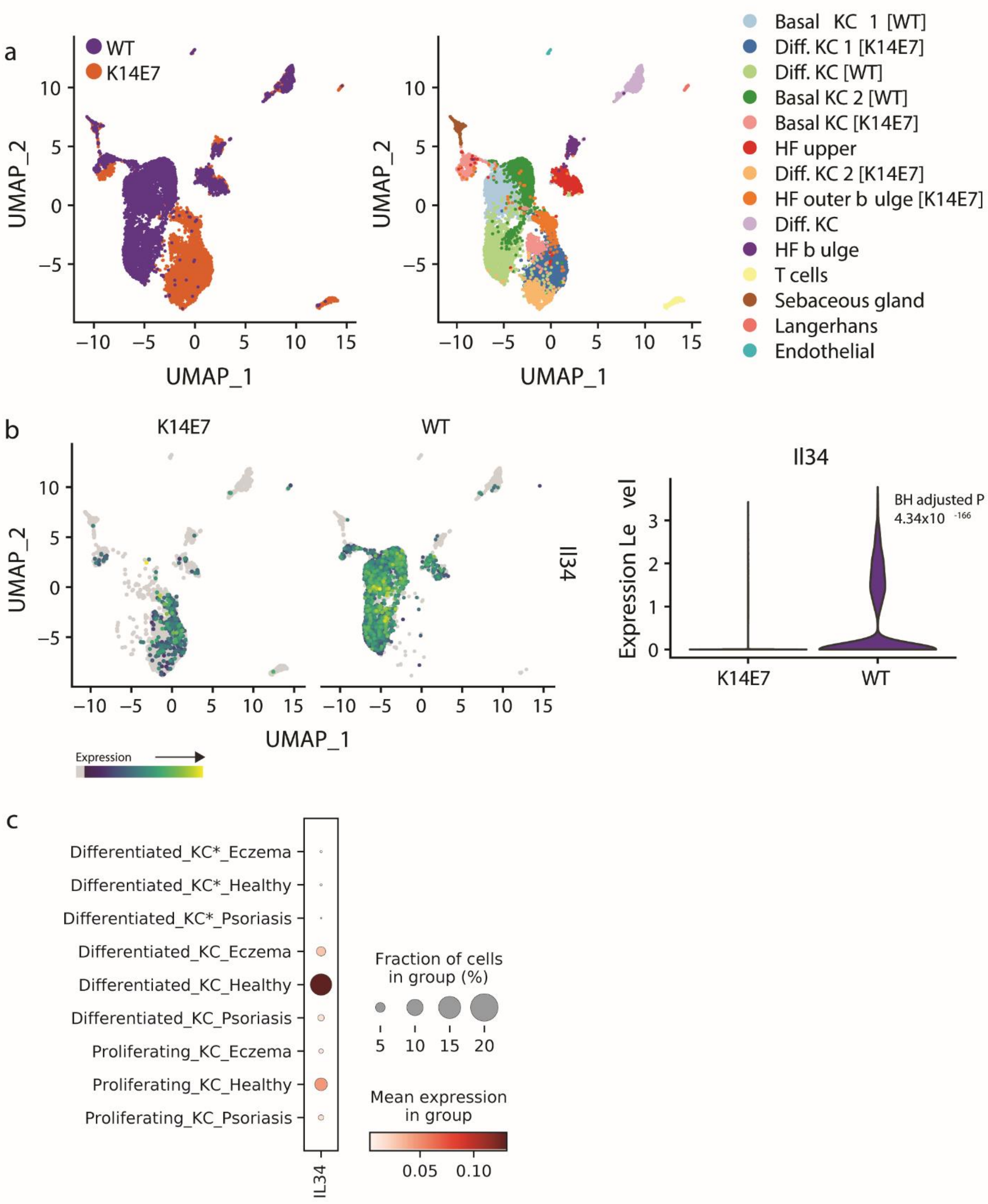

Figure S7. Related to Figure 6.

(a) UMAP plot of KCs from WT and K14E7 skin. Cluster identities were assigned as per previously published. (b) Expression of *Il34* displayed as a heatmap superimposed on UMAP plot (left) of CD45- WT and K14E7 cells and (right) a violin plot (bottom) grouped using only KCs. Differential gene testing was performed between all WT KCs and K14E7 KCs using Wilcoxon Rank Sum test in Seurat's *FindAllMarkers* function and the Benjamini and Hochberg (BH) adjusted p-values for *Il34* is shown. (c) Mean expression dot plot of IL34 in epithelial cells from healthy, psoriasis or eczema human skin. Size of circles indicate percentage of cells expressing the gene (greater than zero), and color gradient corresponds to expression level ranging from white to red for low to high expression across the epithelial clusters.
